# Quantitative phase imaging by gradient retardance optical microscopy

**DOI:** 10.1038/s41598-024-60057-y

**Published:** 2024-04-29

**Authors:** Jinming Zhang, Mirsaeid Sarollahi, Shirley Luckhart, Maria J. Harrison, Andreas E. Vasdekis

**Affiliations:** 1 Department of Physics, University of Idaho, 875 Perimeter Drive, Moscow, ID 83844 USA; 2 Department of Entomology, Plant Pathology and Nematology, University of Idaho, 875 Perimeter Drive, Moscow, ID 83844 USA; 3 Department of Biological Sciences, University of Idaho, 875 Perimeter Drive, Moscow, ID 83844 USA; 4 Boyce Thompson Institute, 533 Tower Rd, Ithaca, NY 14853 USA

**Keywords:** Interference microscopy, Imaging and sensing

## Abstract

Quantitative phase imaging (QPI) has become a vital tool in bioimaging, offering precise measurements of wavefront distortion and, thus, of key cellular metabolism metrics, such as dry mass and density. However, only a few QPI applications have been demonstrated in optically thick specimens, where scattering increases background and reduces contrast. Building upon the concept of structured illumination interferometry, we introduce Gradient Retardance Optical Microscopy (GROM) for QPI of both thin and thick samples. GROM transforms any standard Differential Interference Contrast (DIC) microscope into a QPI platform by incorporating a liquid crystal retarder into the illumination path, enabling independent phase-shifting of the DIC microscope's sheared beams. GROM greatly simplifies related configurations, reduces costs, and eradicates energy losses in parallel imaging modalities, such as fluorescence. We successfully tested GROM on a diverse range of specimens, from microbes and red blood cells to optically thick (~ 300 μm) plant roots without fixation or clearing.

## Introduction

Optical microscopy remains one of the most widespread methods for investigating the physiology of live biosystems^1–9^. Among the various optical microscopy techniques, interferometric or quantitative phase imaging (QPI) stands out for its ability to measure wavefront distortion or optical phase—a key metric that electrodynamics can convert to two important, yet poorly understood, parameters of cellular metabolism: dry-density and dry-mass^10–15^. An additional advantage of QPI is that it is label-free, making it compatible with simpler protocols and imaging at low irradiance levels. The latter sets it apart from fluorescence or Raman imaging, which can suffer from phototoxicity and photobleaching^16,17^. Further, QPI offers relatively increased contrast between cells and some organelles with the background, making it ideal for computation-free cellular and organelle segmentation^18–20^. In a similar context, QPI’s compatibility with AI has opened new avenues towards improved specificity in image classification^21^. As a result, QPI has been widely utilized in exploring the structural and metabolic properties of single, living cells^10–15^, with recent applications extending to super-resolved imaging^22^.

Overall, these advantages make QPI a versatile tool for studying living organisms. However, most QPI applications to date have been on isolated microbial or mammalian cells, with few investigations performed in optically thick specimens, such as multicellular systems or directly in tissue^23–25^. This is because optically thick specimens impose multiple scattering conditions that increase background levels and reduce contrast. As such, performing QPI in multiply scattering environments requires specific strategies, such as combining laser-based tomography^26,27^ with dedicated reconstruction strategies^28^. In similar embodiments, exceptional QPI outcomes in thick specimens were reported through either the backpropagation of partial reconstructions from holograms captured at various angles or the application of specific optical diffraction tomography and image-stitching algorithms that take optical scattering into account^29,30^. Alternative strategies rely on temporally incoherent illumination that—additionally—suppress speckle noise and enable high resolution imaging^31^. The most common theme in these strategies is the use of asymmetric detection or illumination to generate gradient-phase images along a specific axis (x) of the target (∇_x_Φ). In essence, these gradient-phase images represent the first derivative of wavefront distortion of the specimen along the same axis (∇_x_Φ = ∂Φ/∂x) and can, thus, deliver the specimen’s phase map after integration or deconvolution. In this context, structured detection or illumination schemes such as LED and fiber arrays^24,25,32^, differential interference contrast (DIC) imaging^33^, and pupil modifications^34^, have been successfully demonstrated.

DIC-based QPI has attracted considerable attention for its compatibility with standard, commercially available microscopes. In this context, DIC microscopes generate asymmetric illumination from two cross-polarized interfering fields that are created by directing polarized light to a Wollaston (or Savart) prism^35^. Essentially, these two fields form a shear interferometer and are spatially separated by less than the width of the point spread function^36^. Critically, these small shear distances compensate against nonuniformities outside or within the detection volume by ensuring that the two fields suffer equal degradation as they transverse through the specimen^23^. Similar to other asymmetric illumination schemes, DIC imaging yields the gradient-phase (∇_x_Φ) of the specimen, which can be converted directly to wavefront distortion information by Wiener deconvolution^33,37^. More recently, DIC-based QPI was combined with phase shifting interferometry (PSI) to enhance sensitivity and robustness. In this context, PSI was enabled by modifying the imaging path using combinations of quarter waveplates and a rotatable analyzer^38^ or polarization sensitive cameras^39–42^, as well as spatial light modulators in the imaging path^23^. Alternative approaches include specimen rotation^43–45^ or modifying both the illumination and detection paths with a liquid crystal polarization rotator between the Wollaston prisms and polarizers^46^.

**Figure 1:**
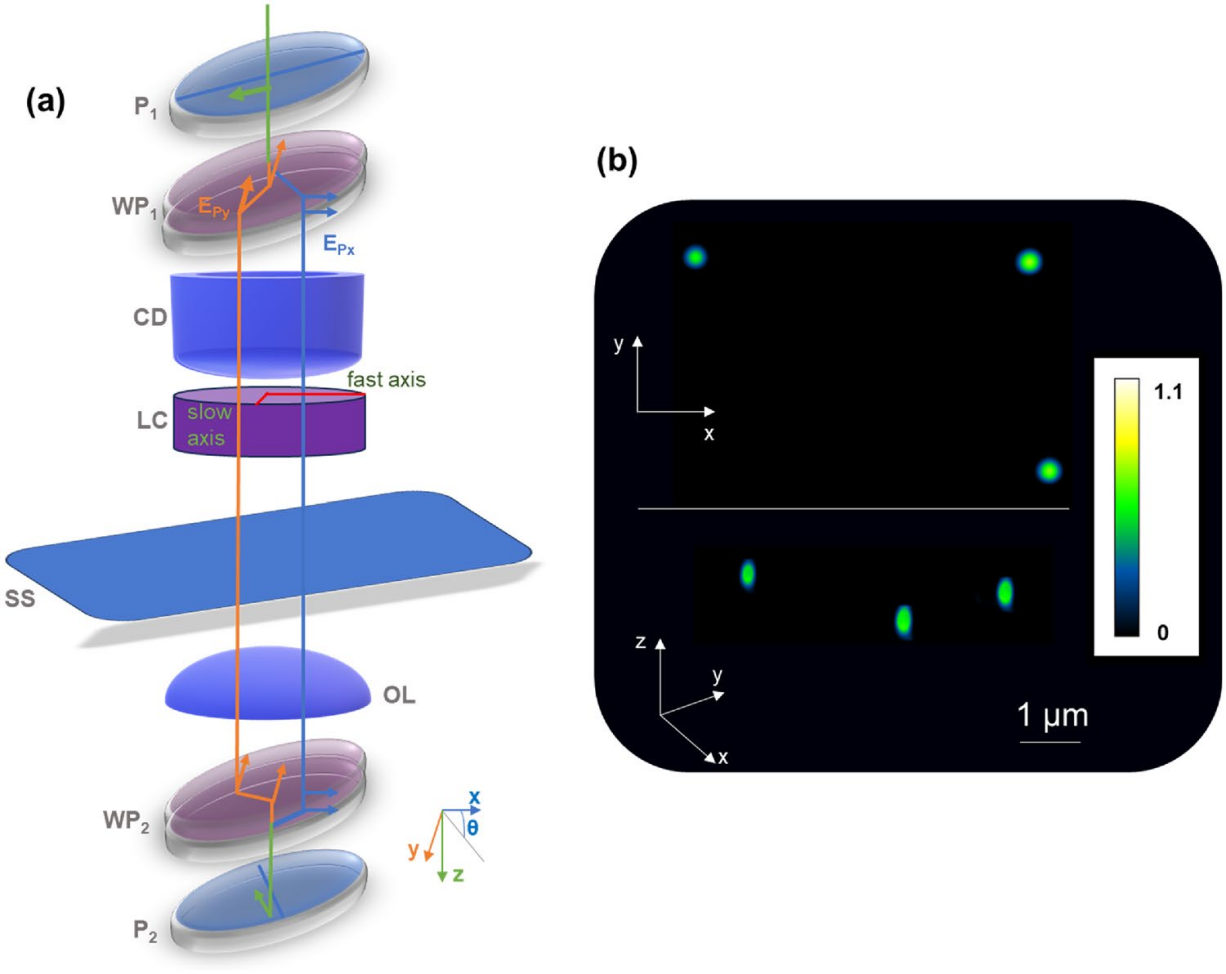
(**a**) GROM illustration combining a liquid–crystal retarder (LC) with a standard differential interference contrast (DIC) microscope that includes polarizers (P1 and P2), Wollaston prisms (WP1 and WP2), condenser (CD), sample (SS) and objective (OL). The optical path illustrates the e- and o-waves generated at WP1 and recombining at WP2. (**b**) An example 3D phase image of 500 nm polystyrene particles immersed in water; scale bar represents the phase delay in radians, which, in this case, depends on the local particle size; the image is composed of a z-stack of 12 layers, each separated by 200 nm.

While these DIC-PSI approaches have been successful in imaging individual cells, cell monolayers, and in some cases optically thick samples^23^, they are not fully congruent with the hardware simplicity conferred by standard DIC microscopes. This is because existing DIC-PSI approaches necessitate several additional hardware components that increase cost and alignment complexity. Further, DIC-PSI approaches that engineer the detection path usually yield considerable energy losses and often require separate detection pathways for fluorescence microscopy. Here, we address these shortcomings by demonstrating a considerably simpler QPI method, termed Gradient Retardance Optical Microscopy (GROM). GROM combines DIC with a liquid crystal retarder that can be placed anywhere between the illumination and detection prisms (Fig. 1a). As such, this combination enables seamless integration with both hardware and open-access software, such as MicroManager^47^. Overall, our approach enables 3D QPI of both optically thin and thick specimens (Fig. 1b), requires a single and cost-effective component, is fully compatible with any standard DIC microscope, and bestows zero energy losses in parallel imaging modalities, such as fluorescence. We demonstrate the utility of our approach by imaging microbial and red blood cells, as well as optically thick (~ 300 μm) embryonic root tissue of the model plant system *Medicago truncatula*.

## Results

### Design and assembly

Our DIC-based QPI system integrates a liquid crystal (LC) retarder into a standard DIC microscope (DMi8, Leica). The DIC microscope operates in a standard Koehler configuration through a relatively high numerical aperture condenser (0.30) and a 7 cm working distance using low coherence illumination centered at 650 nm (20 nm bandwidth) via a bandpass filter (FBH65-20, Thorlabs). We performed DIC imaging by combining two Wollaston prisms (Leica) with a polarizer and analyzer (Leica) in the illumination and detection paths, respectively (Fig. 1a, Fig. S1). As per standard DIC imaging, the first prism splits the incoming light into the two orthogonally polarized beams (e- and o-waves), and the second prism interferes with these beams after they transverse the specimen. We collected images at various magnification levels (10×﻿, 20×﻿, 40×﻿) with the liquid crystal retarder enabling phase-shifting by varying the retardance between the e- and o-waves. In this context, we could place the liquid crystal retarder anywhere between the two prisms (Fig. 1a). In this demonstration, we placed the liquid crystal retarder just after the illumination condenser (Fig. 1a), thus minimizing any modifications to our existing DIC microscope frame.

### Image acquisition

Our approach first required the acquisition of gradient-phase images (∇_x_Φ) and then their conversion to quantitative-phase images (ΔΦ). To acquire the phase-shifted gradient-phase images, we first adjusted the orientation of illumination and detection polarizers and the relative position of the illumination and detection Wollaston prisms (Fig. 1a). We confirmed prism alignment by inspecting both the interference pattern in the Fourier plane via a Bertrand lens and the symmetry of the gradient-phase image of a polystyrene (PS) particle (1 μm in diameter) immersed in oil (n = 1.518, Cargille). The alignment procedure in both the Fourier plane and the gradient-phase image of a polystyrene particle can be visualized through the comparisons presented in Fig. S2. Subsequently, we aligned the liquid crystal (LC) retarder (Meadowlark Optics) using a rotational mount (RSP2, Thorlabs) until its fast axis formed a 45° angle with the illumination polarizer (Fig. 1a). We similarly confirmed this alignment and the calibration of the retarder voltage at the various retardance values (α) by inspecting the shear axis in the Fourier plane (with a Bertrand lens) and symmetry of the gradient-phase image of a PS particle (see comparisons presented in Fig. S3).

### Automation

To fully automate the 3D image acquisition process, we synchronized sample positioning with image capture and the state of the LC retarder. To accomplish this, we combined the open-source microscope control software, MicroManager 2.0, with an Arduino controller (UNO). As depicted in Fig. S4, stage motion (Advanced Scientific Instrumentation) initiated both the image capture through a CMOS camera (Ace acA4024-29um, Basler) and varying voltage levels of the LC retarder. To ensure precise timing of the image acquisition, we set a brief delay (0.1 ms) between setting the LC retarder voltage and capturing the image. This synchronization ensured that specific specimen regions were imaged at preset retardance phases (Δα) between the e- and o-waves. We typically set Δα to obtain uniform retardance variations between 0 and 2π, as illustrated in Fig. S5. Given MicroManager’s widespread use in microscopy, our automation approach can be easily adapted to other microscope and stage configurations.

### Gradient-phase imaging

Generally, for a specimen of amplitude of “J” and phase “θ”, the complex amplitude “U”, of the DIC’s orthogonally polarized e- and o-waves subjected to a pre-sample phase bias “α” (like that from our LC retarder) can be represented as^38^:

1 $${{\text{U}}}_{{\text{e}}}={{\text{J}}}_{{\text{e}}}\cdot {{\text{e}}}^{{\text{i}}{\cdot\uptheta }_{{\text{e}}}},$$

2 $${{\text{U}}}_{{\text{o}}}={{\text{J}}}_{{\text{o}}}{\cdot {\text{e}}}^{{\text{i}}{\cdot (\uptheta }_{{\text{o}}} +\mathrm{ \alpha })}.$$

Under these conditions, the DIC intensity image can be expressed as:

3 $${\text{I}}={{{\text{J}}}_{{\text{o}}}}^{2}+{{{\text{J}}}_{{\text{e}}}}^{2}+2{\cdot {\text{J}}}_{{\text{o}}}\cdot {{\text{J}}}_{{\text{e}}}\cdot {\text{cos}}({\uptheta }_{{\text{e}}}-{\uptheta }_{{\text{o}}}-\mathrm{\alpha }).$$

As such, by capturing phase-shifted intensity images at distinct bias levels of α = 0, π/2, π, and 3·π/2, the specimen’s phase difference (or phase gradient ∇_x_Φ along the gradient direction x) between the e- and o-waves can be expressed as^36^:

4 $${\nabla }_{{\text{x}}}\Phi ={{\text{tan}}}^{-1}\left(\frac{{{\text{I}}}_{\uppi /2}-{{\text{I}}}_{3\uppi /2}}{{{\text{I}}}_{0}-{{\text{I}}}_{\uppi }}\right).$$

Following a similar procedure, one can reconstruct the specimen’s gradient-phase image (∇_x_Φ) using an arbitrary number of bias levels (i) as follows^35^:

5 $${\nabla }_{{\text{x}}}\Phi = - {{\text{tan}}}^{-1}\left[\frac{\sum_{{\text{i}}}{\text{I}}\left({\mathrm{\alpha }}_{{\text{i}}} ,\upphi \right)\cdot \frac{{\text{sin}}\left({\mathrm{\alpha }}_{{\text{i}}}\right)}{\uppi }\cdot \Delta {\mathrm{\alpha }}_{{\text{i}}}}{\sum_{{\text{i}}}{\text{I}}\left({\mathrm{\alpha }}_{{\text{i}}} ,\upphi \right)\cdot \frac{{\text{cos}}\left({\mathrm{\alpha }}_{{\text{i}}}\right)}{\uppi }\cdot \Delta {\mathrm{\alpha }}_{{\text{i}}}}\right].$$

In this application, we configured GROM such that it can collect gradient phase images at 16 bias levels. As further detailed below and discussed elsewhere^35^, we found that 16 bias levels offered enhanced sensitivity and robustness.

### QPI using GROM

**Figure 2:**
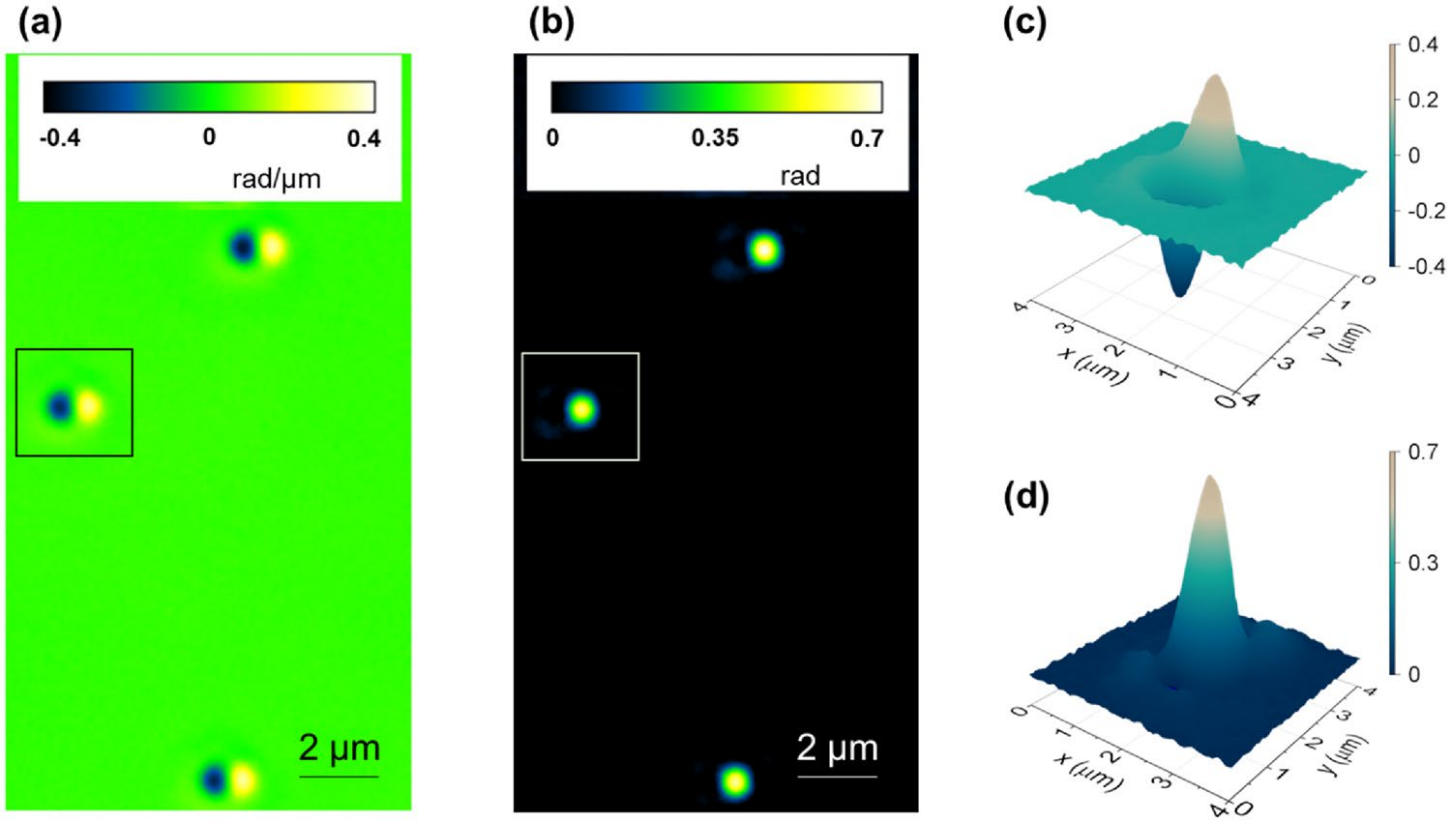
(**a**) Gradient-phase (∇_x_Φ) and (**b**) reconstructed quantitative-phase (ΔΦ) images at 40 × of ~ 1 μm polystyrene particles embedded in immersion oil, with the corresponding 2D maps of an individual particle highlighted by the square in (**c,d**), respectively. (**d**) These data denote a peak phase value of 0.65–0.7 rad, in agreement with the expected value of 0.68 rad.

**Figure 3:**
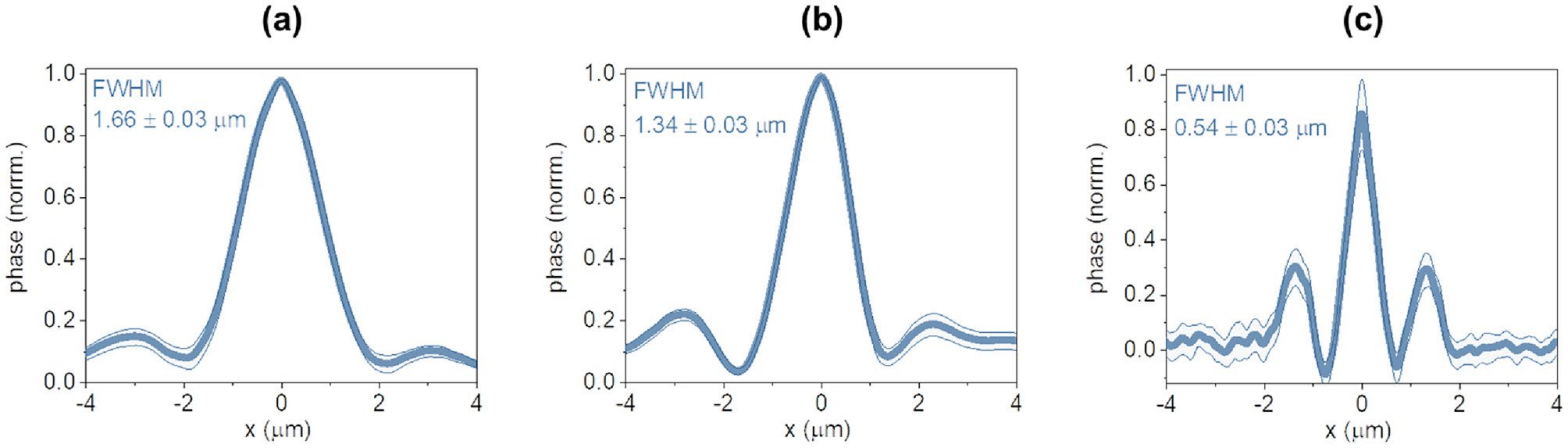
Full-width half maximum (FWHM) of polystyrene particles on a glass coverslip at (**a**) 10× magnification (1 μm diameter particles), (**b**) 20× magnification (1 μm diameter), and (**c**) 40× magnification (200 nm diameter) denoting the planar resolution; blue thick lines depict the experimentally determined mean values and the blue shaded areas the 95% confidence intervals, while the legend notes the average (± s.e.) of n = 20 observations.

To reconstruct the quantitative phase (ΔΦ) images from phase-gradient (∇_x_Φ) ones, we performed an integration operation along the gradient axis as:

6 $$\Phi \left({\text{x}},{\text{y}}\right)= \underset{0}{\overset{{\text{x}}}{\int }}\left[{\nabla }_{{\text{x}}}\Phi \left({{\text{x}}}^{\mathrm{^{\prime}}},{\text{y}}\right)\right]{{\text{dx}}}^{\mathrm{^{\prime}}}+{\text{c}},$$

where “c” is a background constant and equal to Φ (0, y), which can be set to zero if there is no wavefront distortion at the [0, y] coordinate. Several techniques have been deployed in the past to carry out this integration, with Wiener deconvolution gathering most attention^33,37^. In our experiments, however, we found that Wiener deconvolution required curation of several parameters of the point spread function, including the shear distance between the e- and o-waves that needs to be determined experimentally^48^. Even after these adjustments, we found that image reconstruction with Wiener deconvolution was not satisfactory (Fig. S6). In contrast, Hilbert transforms delivered excellent results as demonstrated in Fig. 2 that displays the 3D gradient-phase (∇_x_Φ, Fig. 2a) and quantitative-phase (ΔΦ, Fig. 2b) maps of a 1 μm diameter polystyrene particle immersed in oil (n = 1.518, Cargille). Note that our Hilbert transform results agree with the expected ground truth in terms of the phase amplitude of the particle (see additional comparisons in Supplementary Table I). Consequently, we adopted the Hilbert transform to convert gradient-phase images into quantitative-phase images, followed by background correction (ImageJ)^49^ to render a uniform background. In this configuration, we acquired quantitative-phase images at different magnification levels and imaging fields-of-view as follows (Fig. 3):

- 10×: 744 × 560 μm^2^ FOV and planar resolution of 1.66 ± 0.03 μm (the full width at half maximum of a Gaussian fit of the average trace of n = 20 polystyrene particles with 1 μm diameter immersed in oil, Fig. 3a).
- 20×: 372 × 280 μm^2^ areas and planar resolution of 1.34 ± 0.03 μm (n = 20 particles with 1 μm diameter immersed in oil, Fig. 3b).
- 40×: 186 × 140 μm^2^ and 0.54 ± 0.03 μm (n = 20 particles with 200 nm diameter immersed in water, Fig. 3c).

Further, by scanning the sample with respect to the objective, GROM is capable of 3D QPI imaging. This optical sectioning capability is constrained by the numerical aperture of both the illumination condenser and the detection objective, resulting in 1.63 ± 0.03 μm sectioning resolution specifically for the 40× detection objective (see Fig. S7). Finally, to qualitatively evaluate the sensitivity of our approach, we captured the gradient-phase image of 200 nm diameter particles. As displayed in Fig. S8, 4 phase-shifting steps did not yield adequate sensitivity, rendering the particles invisible. In contrast, incrementing the phase-shifting steps from 4 to 16 enhanced sensitivity, in agreement with findings reported elsewhere^35^.

### GROM bioimaging

**Figure 4:**
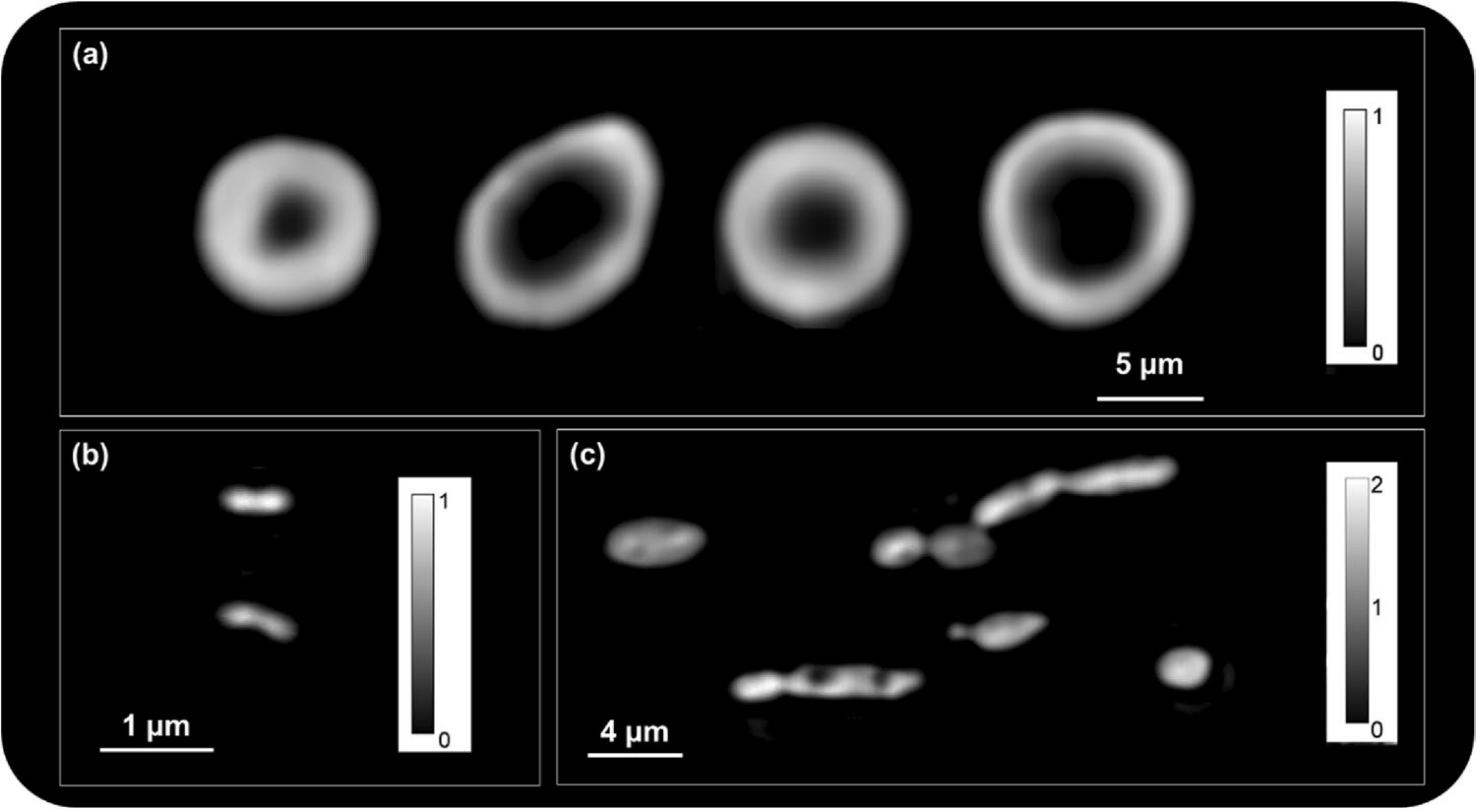
GROM images of red blood cells (**a**), *Escherichia coli* (**b**), and *Yarrowia lipolytica* (**c**). All calibration bars are in radians.

Utilizing the prototype described above, we demonstrated the efficacy of GROM by capturing images of a wide variety of biological specimens, including microbial and red-blood cells. Similar to all QPI methods, by quantifying the specimen’s phase maps, GROM can quantify parameters such as dry-density, mass, cell volume, and area with both cellular and subcellular resolution. For cell biomass, this can be accomplished using the refractive index increment approach, while for intracellular solid objects (e.g., lipid droplets), one can deploy the Clausius Mossotti equation, as we recently reported^15^. Figure 4 displays phase images using a 40×/0.75 NA objective of red-blood cells (a), of mid-exponential *Escherichia coli* microbial cells (b), and *Yarrowia lipolytica* yeast cells (c). Each acquisition required approximately 30 ms per phase-shifting step, leading to as low as 480 ms integration times for a gradient-phase image of 16 phase-shifting steps.

**Figure 5:**
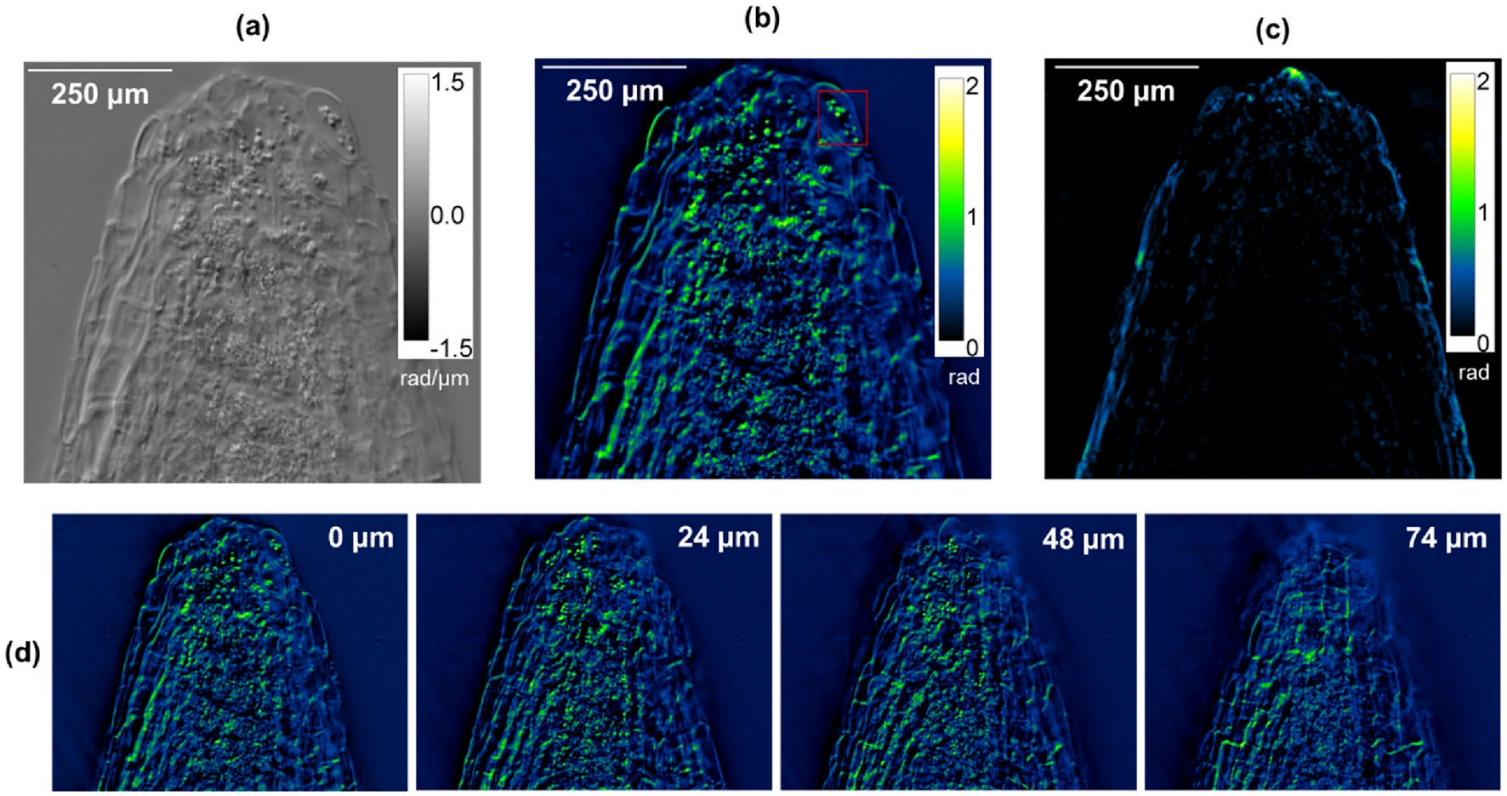
Gradient (**a**) and quantitative (**b**) phase images of a live *Medicago truncatula* root tip using GROM. The corresponding SLIM image of the same root segment is displayed in (**c**). The red square at the root tip in (**b**) indicates the location of starch granules. (**d**) Select z-sections of the image presented in (**b**); *inset* displays the different z-planes relative to the central plane at 0 μm.

We further investigated GROM’s capacity to image optically thick samples by using the live root of a model plant, *Medicago truncatula*. Using a 20×/0.4 NA objective, we captured 3D images by scanning the root axially over a 330 µm range in 3 µm increments. Figure 5a presents the planar view of the gradient-phase image (∇_x_Φ), while Fig. 5b shows the reconstructed phase map (ΔΦ) of the same area. In parallel, we used Spatial Light Interference Microscopy (SLIM)^31^, another QPI method, to image the same root section. As displayed in Fig. 5c, the SLIM image suffers from dark, low information areas, while GROM unveils a plethora of structures in 3D throughout the root’s 270 μm diameter (Fig. 5d). These features include internal cells and their walls, as well as the vascular bundle of the root's core and the starch granules at the tip (highlighted by the red square in Fig. 5b). We attribute the ability to image within optically thick specimens to the minimal lateral shift between the interfering e- and o-waves, which ensures that both fields experience similar degradation due to scattering.

**Figure 6:**
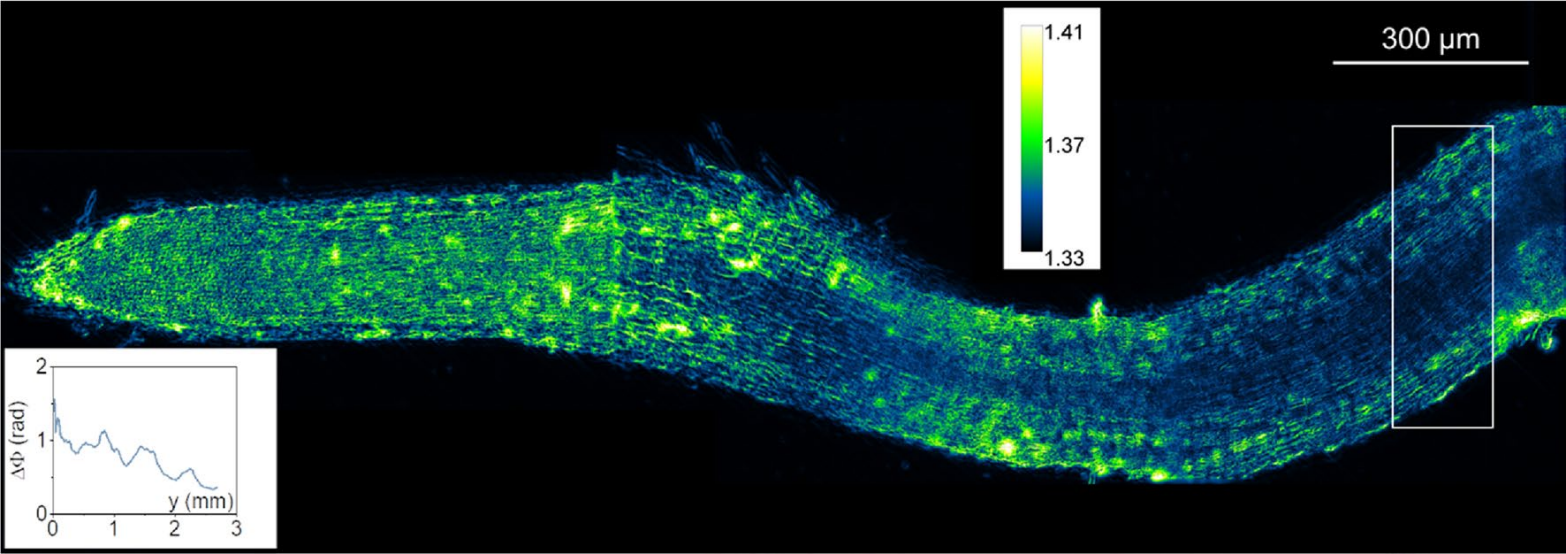
GROM image of a live *Medicago truncatula* root; the image represents a single plane from a 3D stack; scale bar represents the image contrast in refractive index units. At its thickest part (*white rectangle*), the root has a diameter of 325 μm; *inset* plots the radially averaged phase (ΔΦ) from the left to the right.

We further imaged a considerably longer segment (~ 3 mm) of a live *M. truncatula* root using the 10×/0.32 NA objective and the automated area scanning function of MicroManager (Fig. 6). Even at this magnification level, GROM revealed the internal structure of the root, such as the vascular bundle. This investigation also revealed that the root phase increases towards the root tip (Fig. 6, *inset*). This is indicative of larger dry density at the tip and consistent with small and cytoplasmically dense cells in the root meristem. Collectively, our bioimaging exercises suggest that GROM offers a straightforward, cost-effective alternative for imaging both optically thin and thick biological samples.

## Discussion

By operating with two sheared optical beams, standard DIC microscopes have garnered significant attention in shear interferometric bioimaging^35^. Compared to laser-based tomographic methods, which provide exceptional sensitivity and allow for numerical adjustment of the focal plane, DIC microscopy-based approaches utilize incoherent illumination. As a result, DIC approaches can suppress speckle, while they do not require numerical backpropagation computations^20,28–30^. In the context of QPI using commercially available DIC microscope frames, earlier attempts deployed Wiener deconvolution to convert DIC images to quantitative-phase maps^33^. Despite offering promising results, this approach can be suboptimal by requiring the adjustment of several deconvolution parameters. More recently, DIC shear interferometry was combined with phase-shifting for increased sensitivity and fewer image ambiguities^23,46^. In this context, a quarter wave plate (QWP) was introduced between the Wollaston prism and analyzer in the detection path^38,44^. In this case, the QWP converted the e- and o-DIC waves after the specimen to oppositely handed circular polarizations. By manually rotating the analyzer, it was possible to precisely control phase shifting, albeit at inevitably reduced imaging rates. More recently, others relegated the need for manual intervention by replacing the rotatable analyzer with a polarization sensitive camera that, however, decreased resolution and increased costs by requiring additional detectors for fluorescence imaging^41^.

Other DIC-based PSI methods have been successfully demonstrated by requiring the mechanical rotation of the specimen with respect to the shear axis^45,46^. These approaches, however, come with the challenge of possible imaging artifacts due to mechanical positioning errors and misalignment, higher costs due to the implementation of a rotational stage, and low imaging rates. In an alternative embodiment, complex assemblies consisting of a liquid crystal retarder between two Wollaston prisms at both the illumination and detection paths enabled both the rotation of the shear axis and phase-shifting. While successful, this approach suffered from key practical challenges, including the need for alignment of complex additional hardware components that are not congruent with the simplicity conferred by standard DIC microscopes. In another embodiment, the DIC image was transferred to a spatial light modulator (SLM) to vary the phase-delay between the sheared beams^23^. Critically, this approach enabled 3D phase imaging in tissue (250 μm thick bovine embryos); however, GLIM inevitably suffers from energy losses and high costs, as both the SLM and analyzer are integral to the imaging path^23^.

To overcome these shortcomings, we introduced an alternative approach that places a LC retarder between the Wollaston prisms of a standard DIC microscope (Fig. 1a). In this way, the retarder shears the e- and o-waves at precisely controlled phase-shifting levels *before* they illuminate the sample. By deploying a single optical element in the illumination path, our approach greatly simplifies related hardware configurations and incurs no energy losses in alternative detection modes. Compared to laser-based QPI techniques^29,30^, GROM operates independently of any need to characterize or make assumptions about the scattering medium, though it may exhibit lower sensitivity. To address this limitation, we have developed a method that involves capturing additional images at finer differences in retardance (see Fig. S8), which allows GROM to enhance sensitivity. Further, the integration of a single and cost-effective element with open-access control software, and the possibility of using the same camera sensor for both quantitative-phase and fluorescence imaging not only minimizes losses, but also the overall cost of the assembly. To demonstrate the validity of our approach, we successfully imaged a wide variety of samples, including polymer particles, bacteria and red-blood cells, as well as optically thick tissue samples. As such, our approach addresses a longstanding challenge in QPI to provide insights into multicellular systems in a cost-effective manner without the need for fixation or clearing.

## Methods

All particles (1 μm, 500 nm, and 200 nm diameters) used in this study were purchased from Bangs Laboratories, diluted by approximately 1000×, and let to dry on glass coverslips in a convection oven (50 °C) for 30–60 min. Prior to imaging, the particles were covered with a drop (~ 5 μL) of immersion oil or water and a second glass coverslip. The DH5α *E. coli* strain used in this study was cultured in a standard Mueller Hinton broth (Becton Dickinson) at 37 °C in a shaking incubator at 180 rpm^50^. The MYL035 *Y. lipolytica* strain was cultured in standard YPD rich medium at 28°C in a shaking incubator also at 180 rpm. For the rich YPD medium, we mixed 20 g/L Bacto Peptone (Becton Dickinson), 10 g/L yeast extract (Alfa Aesar), and 20 g/L glucose (Fisher). Both *E. coli* and *Y. lipolytica* precultures were stored in Mueller Hinton and YPD agarose (Invitrogen) plates at 4 °C, respectively, and were passed twice in their respective liquid media (5 mL round bottom polystyrene tubes) for 24 h. Prior to experimentation, cells were passed again at a 0.01–0.02 optical density (OD600, λ = 600 nm, V-1200 spectrometer, VWR) and sampled at the mid-exponential growth stage. Red blood cells were purchased from the Interstate Blood Bank and fixed in 2% paraformaldehyde, followed by 3× washing in 10× PBS prior to imaging. For imaging, we placed the *Y. lipolytica* and red-blood cells between two glass coverslips and pressed gently to minimize cell motion. For *E. coli* cells, we first deposited a small culture volume (1 μL) on a thin (~ 100 μm) agarose (Invitrogen) gel and covered with a glass coverslip, prior to imaging. We prepared root samples by following the procedure detailed here^51^, namely: scarification of *M. truncatula* seeds (sandpaper), sterilization (5 min soaking in 30% Clorox + 0.1% Tween 20), washing (3 × in sterile ddH_2_O), and spreading on sterile filter paper in a petri dish wrapped with parafilm. Subsequently, we kept the seeds at 4 °C for 3 days, then at room temperature first in dark (1 day) and then under light (1000 lx) for growth, before transferring them on a glass coverslip and covering them with low melting point agarose (Thermo Fisher).

### Supplementary Information

Supplementary Information.

## Data Availability

The datasets generated and/or analyzed during the current study are available from the corresponding author upon reasonable request.

## References

[1] Lichtman JW, Conchello J-A (2005). Fluorescence microscopy. Nat. Methods.

[2] Balasubramanian H, Hobson CM, Chew T-L, Aaron JS (2023). Imagining the future of optical microscopy: Everything, everywhere, all at once. Commun. Biol..

[3] Park J, Brady DJ, Zheng G, Tian L, Gao L (2021). Review of bio-optical imaging systems with a high space-bandwidth product. Adv. Photon..

[4] Choi M, Kwok SJJ, Yun SH (2015). In vivo fluorescence microscopy: Lessons from observing cell behavior in their native environment. Physiology.

[5] Herman B, Lemasters JJ (2012). Optical Microscopy: Emerging Methods and Applications.

[6] Weber M, Huisken J (2021). Multidisciplinarity is critical to unlock the full potential of modern light microscopy. Front. Cell Dev. Biol..

[7] Parodi V, Jacchetti E, Osellame R, Cerullo G, Polli D, Raimondi MT (2020). Nonlinear optical microscopy: From fundamentals to applications in live bioimaging. Front. Bioeng. Biotechnol..

[8] Lee K, Kim K, Jung J, Heo J, Cho S, Lee S, Chang G, Jo Y, Park H, Park Y (2013). Quantitative phase imaging techniques for the study of cell pathophysiology: From principles to applications. Sensors.

[9] Wu Y, Rivenson Y, Wang H, Luo Y, Ben-David E, Bentolila LA, Pritz C, Ozcan A (2019). Three-dimensional virtual refocusing of fluorescence microscopy images using deep learning. Nat. Methods.

[10] Marquet P, Depeursinge C, Magistretti PJ (2014). Review of quantitative phase-digital holographic microscopy: Promising novel imaging technique to resolve neuronal network activity and identify cellular biomarkers of psychiatric disorders. Neurophotonics.

[11] Mir M, Bhaduri B, Wang R, Zhu R, Popescu G (2012). Quantitative phase imaging. Prog. Opt..

[12] Park Y, Depeursinge C, Popescu G (2018). Quantitative phase imaging in biomedicine. Nat. Photon..

[13] Popescu G, Park Y, Lue N, Best-Popescu C, Deflores L, Dasari RR, Feld MS, Badizadegan K (2008). Optical imaging of cell mass and growth dynamics. Am. J. Physiol. Cell Physiol..

[14] Barer R (1953). Determination of dry mass, thickness, solid and water concentration in living cells. Nature.

[15] Vasdekis AE, Alanazi H, Silverman AM, Williams CJ, Canul AJ, Cliff JB, Dohnalkova AC, Stephanopoulos G (2019). Eliciting the impacts of cellular noise on metabolic trade-offs by quantitative mass imaging. Nat. Commun..

[16] Lippincott-Schwartz J, Altan-Bonnet N, Patterson GH (2003). Photobleaching and photoactivation: Following protein dynamics in living cells. Nat. Cell Biol..

[17] Hoebe RA, van Oven CH, Gadella TWJ, Dhonukshe PB, van Noorden CJF, Manders EMM (2007). Controlled light-exposure microscopy reduces photobleaching and phototoxicity in fluorescence live-cell imaging. Nat. Biotechnol..

[18] Alanazi H, Canul AJ, Garman A, Quimby J, Vasdekis AE (2017). Robust microbial cell segmentation by optical-phase thresholding with minimal processing requirements. Cytometry A.

[19] Nguyen TH, Kandel M, Shakir HM, Best-Popescu C, Arikkath J, Do MN, Popescu G (2017). Halo-free phase contrast microscopy. Sci. Rep..

[20] Pirone D, Lim J, Merola F, Miccio L, Mugnano M, Bianco V, Cimmino F, Visconte F, Montella A, Capasso M, Iolascon A, Memmolo P, Psaltis D, Ferraro P (2022). Stain-free identification of cell nuclei using tomographic phase microscopy in flow cytometry. Nat. Photon..

[21] Park J, Bai B, Ryu D, Liu T, Lee C, Luo Y, Lee MJ, Huang L, Shin J, Zhang Y, Ryu D, Li Y, Kim G, Min H, Ozcan A, Park Y (2023). Artificial intelligence-enabled quantitative phase imaging methods for life sciences. Nat. Methods.

[22] Cui G, Liu Y, Zu D, Zhao X, Zhang Z, Kim DY, Senaratne P, Fox A, Sept D, Park Y, Lee SE (2023). Phase intensity nanoscope (PINE) opens long-time investigation windows of living matter. Nat. Commun..

[23] Nguyen TH, Kandel ME, Rubessa M, Wheeler MB, Popescu G (2017). Gradient light interference microscopy for 3D imaging of unlabeled specimens. Nat. Commun..

[24] Ledwig P, Robles FE (2019). Epi-mode tomographic quantitative phase imaging in thick scattering samples. Biomed. Opt. Express.

[25] Ford TN, Chu KK, Mertz J (2012). Phase-gradient microscopy in thick tissue with oblique back-illumination. Nat. Methods.

[26] Yoon J, Kim K, Park H, Choi C, Jang S, Park Y (2015). Label-free characterization of white blood cells by measuring 3D refractive index maps. Biomed. Opt. Express.

[27] Jin D, Zhou R, Yaqoob Z, So PTC (2017). Tomographic phase microscopy: Principles and applications in bioimaging [invited]. J. Opt. Soc. Am. B.

[28] Lim J, Ayoub AB, Antoine EE, Psaltis D (2019). High-fidelity optical diffraction tomography of multiple scattering samples. Light Sci. Appl..

[29] Yasuhiko O, Takeuchi K (2023). In-silico clearing approach for deep refractive index tomography by partial reconstruction and wave-backpropagation. Light Sci. Appl..

[30] Hugonnet H, Kim YW, Lee M, Shin S, Hruban RH, Hong S-M, Park Y (2021). Multiscale label-free volumetric holographic histopathology of thick-tissue slides with subcellular resolution. Adv. Photon..

[31] Wang Z, Millet L, Mir M, Ding H, Unarunotai S, Rogers J, Gillette MU, Popescu G (2011). Spatial light interference microscopy (SLIM). Opt. Express.

[32] Tian L, Waller L (2015). Quantitative differential phase contrast imaging in an LED array microscope. Opt. Express.

[33] Van Munster EB, Van Vliet LJ, Aten JA (1997). Reconstruction of optical pathlength distributions from images obtained by a wide-field differential interference contrast microscope. J. Microsc..

[34] Lu H, Chung J, Ou X, Yang C (2016). Quantitative phase imaging and complex field reconstruction by pupil modulation differential phase contrast. Opt. Express.

[35] Terborg RA, Pello J, Mannelli I, Torres JP, Pruneri V (2016). Ultrasensitive interferometric on-chip microscopy of transparent objects. Sci. Adv..

[36] Malacara D (2007). Wiley Series in Pure and Applied Optics.

[37] Jansson PA (1997). Deconvolution of Images and Spectra.

[38] Cogswell CJ, Smith NI, Larkin KG, Hariharan P, Cogswell CJ, Conchello J-A, Wilson T (1997). Quantitative DIC microscopy using a geometric phase shifter. Three-Dimensional Microscopy: Image Acquisition and Processing IV.

[39] Takano W, Shibata S, Hagen N, Matsuda M, Otani Y (2020). Minimizing scattering-induced phase errors in differential interference contrast microscopy. J. Biomed. Opt..

[40] Strassberg M, Shevtsova Y, Kamel D, Wagoner-Oshima K, Zhong H, Xu M (2021). Single-shot quantitative phase imaging with polarization differential interference contrast. Appl. Phys. Lett..

[41] Shibata S, Takano W, Hagen N, Matsuda M, Otani Y (2019). Video-rate quantitative phase analysis by a DIC microscope using a polarization camera. Biomed. Opt. Express.

[42] Yasuhiko O, Takeuchi K, Yamada H, Ueda Y (2021). Single-shot quantitative phase imaging as an extension of differential interference contrast microscopy. Genes Cells.

[43] Preza C (2000). Rotational-diversity phase estimation from differential-interference-contrast microscopy images. J. Opt. Soc. Am. A.

[44] King SV, Libertun A, Piestun R, Cogswell CJ, Preza C (2008). Quantitative phase microscopy through differential interference imaging. J. Biomed. Opt..

[45] Shribak M, Inoue S (2006). Orientation-independent differential interference contrast microscopy. Microsc. Microanal..

[46] Shribak M, Larkin KG, Biggs D (2017). Mapping optical path length and image enhancement using quantitative orientation-independent differential interference contrast microscopy. J. Biomed. Opt..

[47] Edelstein AD, Tsuchida MA, Amodaj N, Pinkard H, Vale RD, Stuurman N (2014). Advanced methods of microscope control using μmanager software. J. Biol. Methods.

[48] Chiu HC, Zeng Z, Zhao L, Zhao T, Du S, Chen X (2019). Measuring optical beam shear angle of polarizing prisms beyond the diffraction limit with localization method. Opt. Commun..

[49] Schneider CA, Rasband WS, Eliceiri KW (2012). NIH Image to ImageJ: 25 years of image analysis. Nat. Methods.

[50] Nemati S, Singh A, Dhuey SD, McDonald A, Weinreich DM, Vasdekis AE (2022). Density fluctuations, homeostasis, and reproduction effects in bacteria. Commun. Biol..

[51] Harrison MJ (1993). Isoflavonoid accumulation and expression of defense gene transcripts during the establishment of vesicular-arbuscular mycorrhizal associations in roots of *Medicago truncatula*. Mol. Plant Microbe Interact..

