# Supplementary material for: Quantitative phase imaging by gradient retardance optical microscopy
Source: Sci Rep. 2024 Apr 29;14:9754. doi: 10.1038/s41598-024-60057-y (PMC11056386; doi:10.1038/s41598-024-60057-y)
Supplement: Supplementary file 1 — Supplementary Information. [file 41598_2024_60057_MOESM1_ESM.pdf]

Supplementary Figures for:

Quantitative Phase Imaging by Gradient Retardance Optical Microscopy

Jinming Zhang[1], Mirsaeid Sarollahi[1], Shirley Luckhart[2,3], Maria J. Harrison[4], Andreas E. Vasdekis[1,*]

[1] Department of Physics, 875 Perimeter Drive, University of Idaho, Moscow, ID, 83844, USA.

[2] Department of Entomology, Plant Pathology and Nematology, 875 Perimeter Drive, University of Idaho, Moscow, ID, 83844, USA.

[3] Department of Biological Sciences, 875 Perimeter Drive, University of Idaho, Moscow, ID, 83844, USA.

[4] Boyce Thompson Institute, 533 Tower Rd., Ithaca, NY, 14853, USA

Figure S1

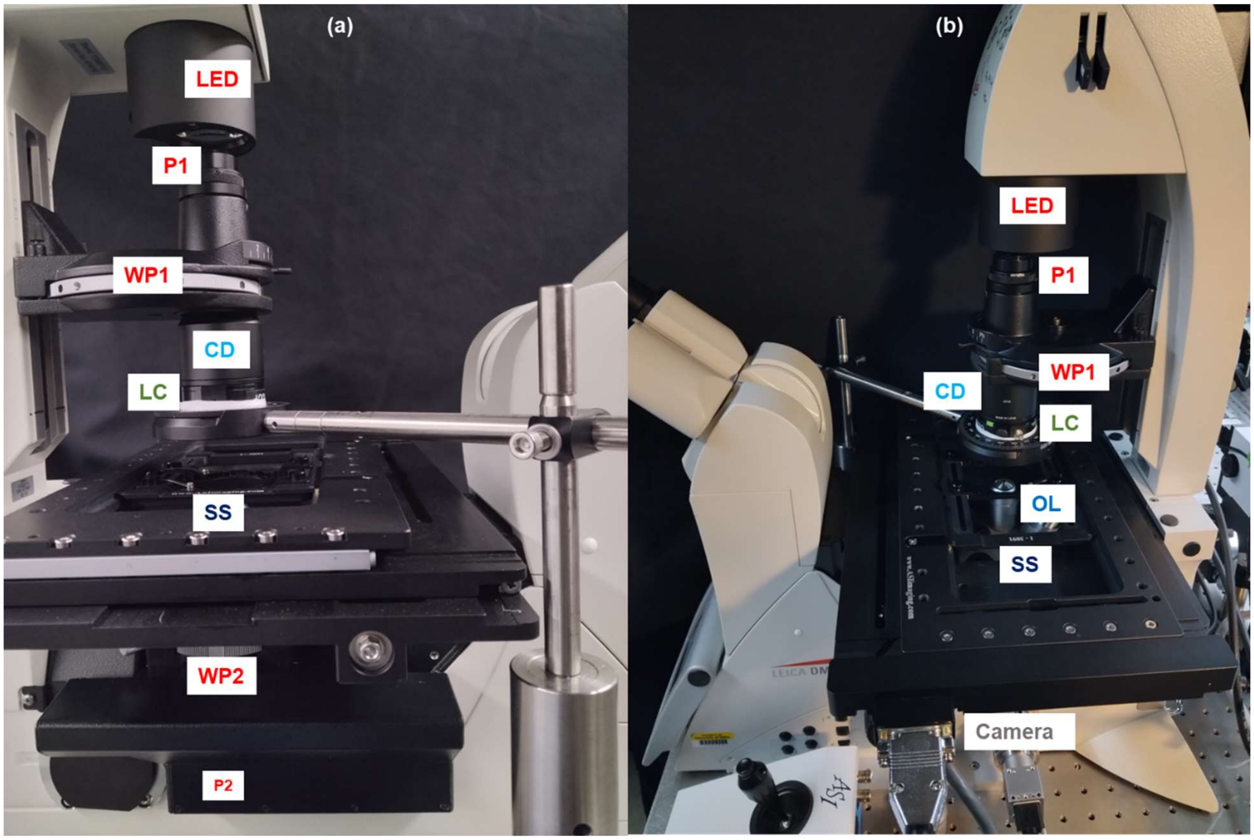

Fig. S1: Left **(a)** and right **(b)** side views of the GROM configuration integrated with a standard DIC microscope and a LC module retarder, including its core optical elements (*P1,2*: polarizers; *WP1,2*: Wollaston prisms; *CD*: Condenser; *DIC*: Differential Interference Contrast; *LC*: Liquid Crystal retarder; *SS*: Sample stage; *OL*: Objective Lens).

Figure S2

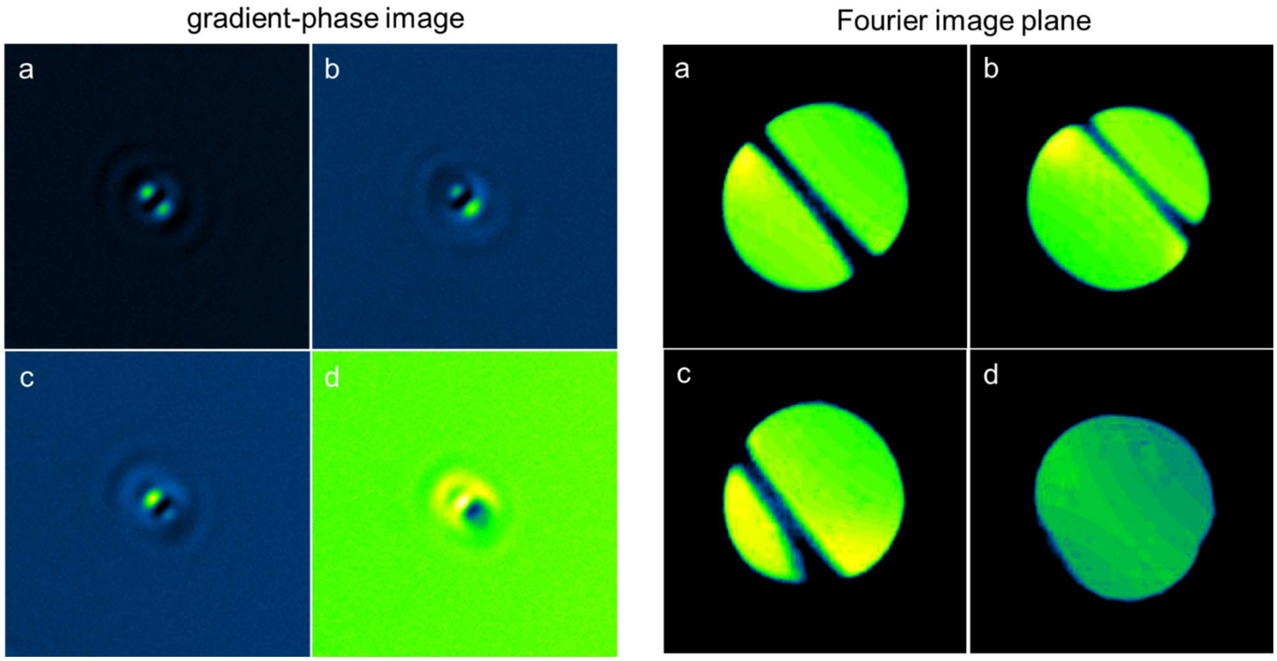

Fig. S2: Prism alignment procedure in real-space gradient-phase images of a 1 μm diameter polystyrene particle (*left*) and in the Fourier imaging plane using a Bernard lens (*right*); in both cases, “a” represents the aligned state, while b-d, the misaligned ones.

Figure S3

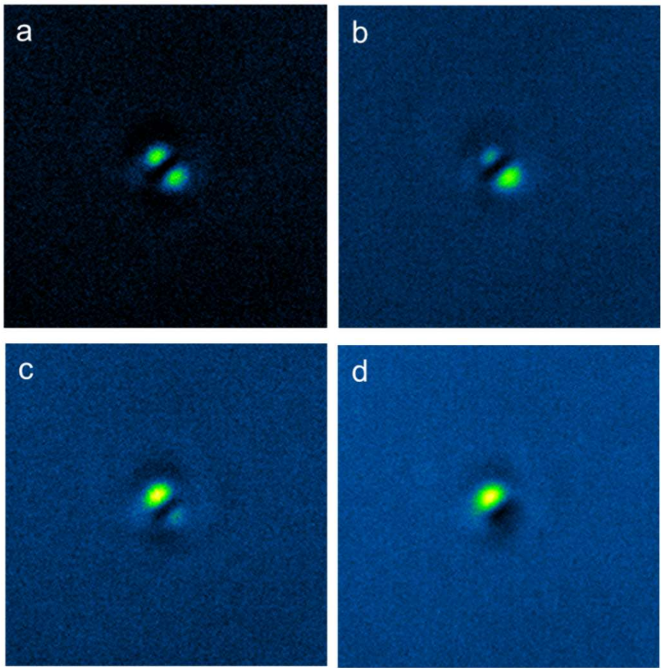

Fig. S3: LC retarder voltage calibration procedure using the gradient-phase images of a 1 μm diameter polystyrene particle; "a" represents the appropriate voltage for retardance values of $\alpha = 0$, while b-d do not.

Figure S4

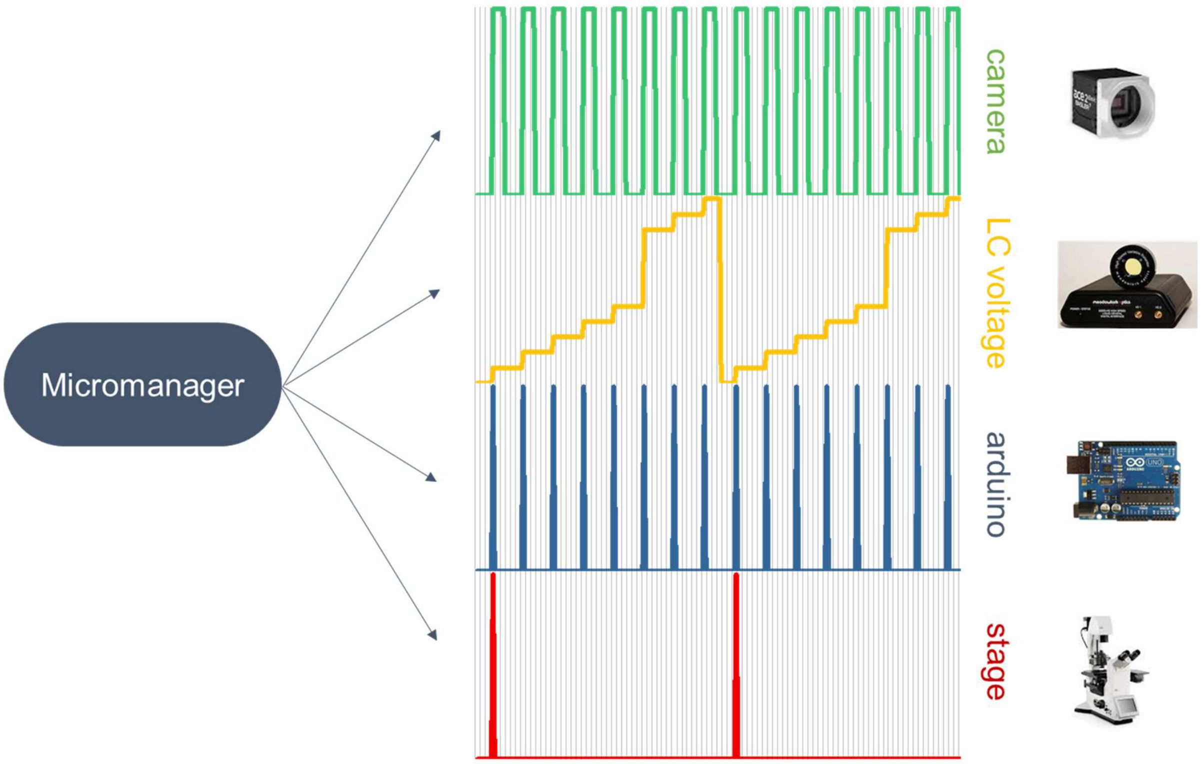

Fig. S4: The synchronization scheme for the camera, LC retarder, arduino device and microscope stage.

Figure S5

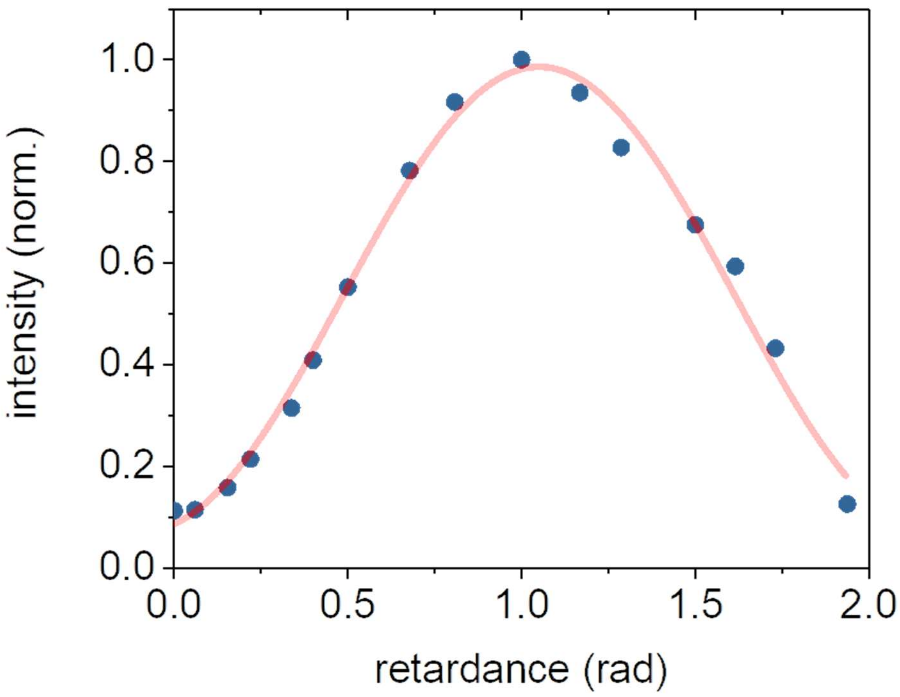

Fig. S5: The background intensity curve at a [0,2Pi] range of retardance (α); red line represents a sinusoidal fit.

Figure S6

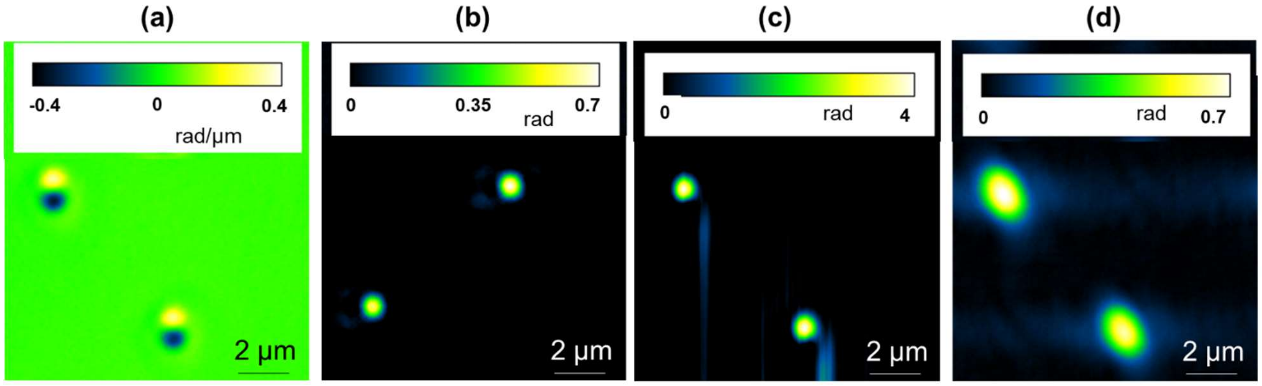

Fig. S6: The reconstruction of the original gradient-phase image **(a)** compared with Hilbert transforms **(b)**, direct integration using the *cumulative sum* command in Matlab **(c)** and Wiener deconvolution **(d)**.

Figure S7

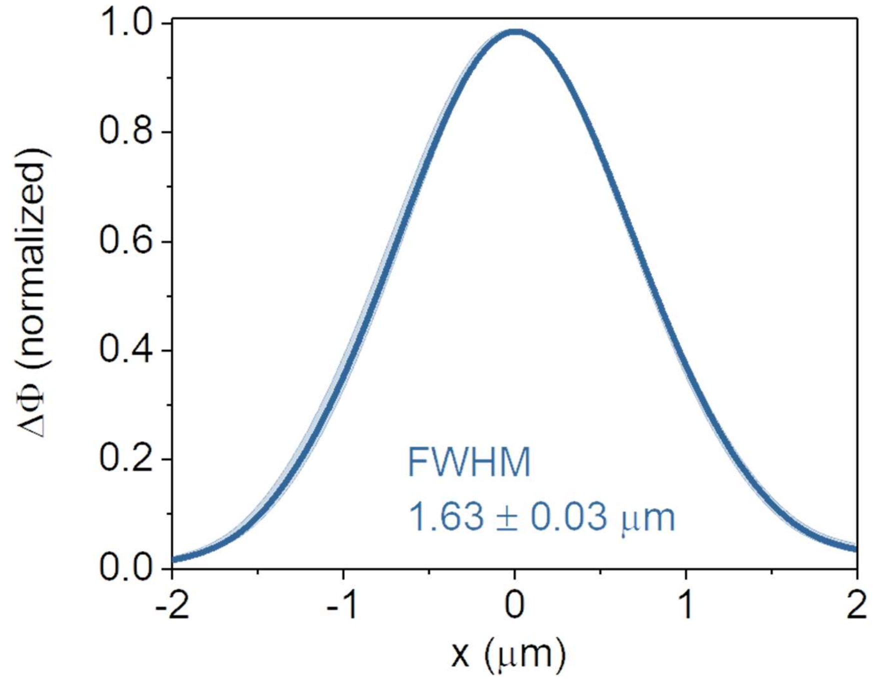

Fig. S7: GROM's optical sectioning capability quantified using 200 nm polystyrene particles and the 40× magnification objective; thick blue lines depict the experimentally determined mean values and the light-blue shaded areas the 95% confidence intervals, while the legend notes the average (± s.e.) of 20 observations.

Figure S8

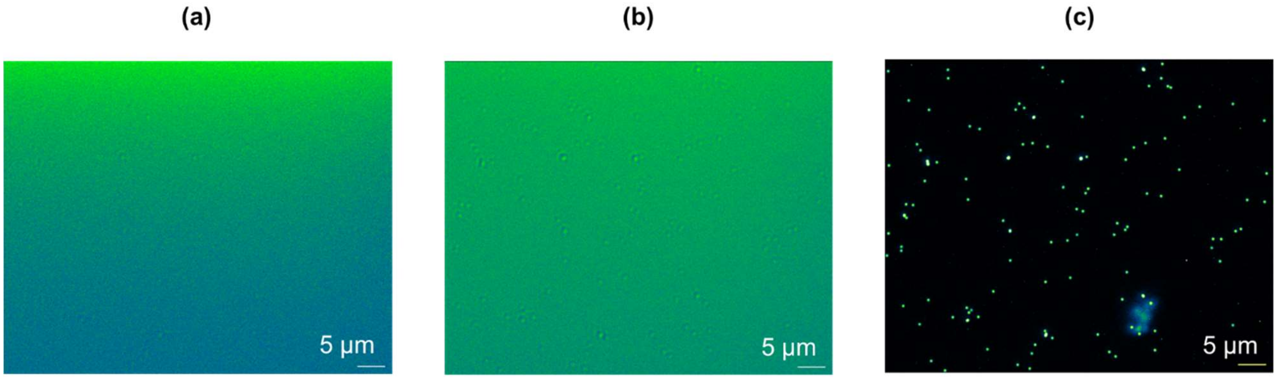

Fig. S8: The QPI images of 200 nm polystyrene particles immersed in oil using 4 **(a)** and 16 **(b)** phase-shifting steps (Δα). The corresponding fluorescence ground truth is displayed in **(c)**.

Supplementary Table I

material	immersion media	diameter (μm)	expected ΔΦ (rad)	measured ΔΦ (rad)
PS	oil	1	0.73	0.74
PMMA	oil	2.5	0.53	0.53
PS	water	0.5	1.24	1.24

Supplementary Table I: Comparison of the measured phase values of certain polymer particles with the corresponding expected values.
